# Efficacy of 2% ganciclovir eye drops in the treatment of cytomegalovirus anterior uveitis

**DOI:** 10.1007/s10384-025-01250-y

**Published:** 2025-08-09

**Authors:** Yu Yoneda, Yuki Takenaka, Nanae Taniguchi, Keisuke Yoneda, Kyosuke Seki, Tomoyuki Oyama, Masaya Imazeki, Masaru Takeuchi

**Affiliations:** Department of Ophthalmology, National Defense Medical College, 3-2 Namiki, Tokorozawa, Saitama 359-8513 Japan

**Keywords:** Anterior uveitis, Cytomegalovirus, Ganciclovir, Herpetic uveitis, Infectious uveitis

## Abstract

**Purpose:**

To investigate the efficacy and safety of 2% ganciclovir (GCV) eye drops for the treatment of primary cytomegalovirus anterior uveitis (CMV-AU)

**Study design:**

Retrospective cohort study

**Methods:**

This study included 12 patients diagnosed with CMV-AU who were treated with 2% GCV eye drops. The patients’ demographics, clinical presentations, treatment regimens, and outcomes were analyzed.

**Results:**

The cohort consisted predominantly of men (11:1 ratio), with a mean age of 63.4 years and all presenting with unilateral disease. Common presenting symptoms were blurred vision and elevated intraocular pressure (IOP). After the initiation of 2% GCV eye drops, all the patients demonstrated positive responses, with improvement in BCVA, decreased IOP, and resolution of keratic precipitates including coin-shaped lesions. Recurrence of uveitis occurred in 66.7% of the patients and was managed with intensified topical corticosteroids, antiglaucoma medications, and/or short-term oral GCV. IOP significantly decreased after treatment (*P* <.05), whilst BCVA and corneal endothelial cell counts remained stable. No patients developed bullous keratopathy or required intravenous GCV. One patient underwent trabeculectomy for uncontrolled IOP.

**Conclusion:**

This study’s findings suggest that 2% GCV eye drops are a safe and effective treatment option for primary CMV-AU, offering improvements in IOP and uveitis control. All the patients completed the treatment without serious adverse events, supporting the favorable safety profile of 2% GCV eye drops.

## Introduction

Although cytomegalovirus (CMV) was once primarily associated with posterior retinitis in immunocompromised individuals, such as those with HIV/AIDS, recent research has shown that CMV can also cause anterior uveitis in immunocompetent hosts [1–4].

CMV anterior uveitis (CMV-AU) has a particularly high prevalence in Asia, especially among men [5]. CMV-AU is most common in those aged 30 to 50 years, often presenting with elevated intraocular pressure (IOP) and mild anterior chamber findings [6]. Chronic CMV-AU has a high prevalence in individuals aged 50 to 70 years and typically presents with ocular discomfort and blurred vision [5]. Especially with inflammation in the pupillary area, atrophy of the iris stroma occurs [3], sometimes accompanied by heterochromia iridis [7], and the symptoms are similar to Fuchs uveitis syndrome (FUS). The diagnosis of CMV-AU can be challenging if based solely on the clinical ophthalmic findings, and before the availability of polymerase chain reaction (PCR) testing using aqueous humor (AH), especially comprehensive PCR testing, many of these cases were diagnosed as Posner-Schlossman syndrome (PSS) or FUS [8, 9].

Delayed diagnosis can result in irreversible damage to the corneal endothelium, leading to endothelial dysfunction and corneal edema, and may ultimately require corneal transplantation [10–13]. Additionally, there is a risk of CMV recurrence in the transplanted cornea, leading to the need for further surgeries and potential complications [14–18]. Furthermore, sustained high IOP can lead to progression of glaucoma, and if left untreated, can result in blindness [19–21]. Prompt antiviral therapy can substantially decrease the likelihood of glaucoma surgery. Early detection and treatment are therefore essential for a favorable outcome.

Oral valganciclovir is an option; however, its high cost and potential for systemic adverse effects including bone marrow suppression and renal impairment necessitate regular blood monitoring. For topical treatment, ganciclovir (GCV) eye drops are typically formulated with concentrations ranging from 0.15% to 2% [22, 23]. The recommended dosing regimen involves instillation 6 to 8 times daily during the initial treatment phase, followed by 1 to 4 times daily for maintenance. According to Langston and colleagues, more frequent administration of GCV gel (every 2 hours) resulted in better efficacy [24]. Antoun's research indicated that 0.15% GCV gel offered the highest therapeutic efficacy. However, patient adherence to this treatment regimen was suboptimal. In contrast, treatment with 2% GCV eye drops demonstrated effective outcomes in patients with CMV retinitis and anterior uveitis [25]. Studies have shown that 2% GCV eye drops provide favorable therapeutic outcomes for patients with CMV-AU, demonstrating advantages in terms of safety, cost-effectiveness, and ease of use. In the current study, we retrospectively investigated the background, ocular findings, and clinical course of patients diagnosed with CMV-AU and treated with 2% GCV eye drops.

## Patients and methods

The clinical records of 12 patients diagnosed with CMV-AU and treated with 2% GCV eye drops between October 2020 and April 2024 at the National Defense Medical College Hospital were retrospectively reviewed. The patients’ demographics, past ophthalmologic and internal medicine histories, reasons for referral to the hospital, best-corrected visual acuity (BCVA) and IOP at the initial and last visits, ocular findings, complications, types of eye drops and oral medications used for treatment, presence or absence of recurrence after the start of treatment, and history of ophthalmic surgery were investigated. For statistical analysis, VA was calculated as 0.0025 for counting fingers, 0.002 for hand motions, 0.0016 for light perception, and 0.0013 for no light perception [26]. The study was conducted in accordance with the Declaration of Helsinki and was approved by the National Defense Medical College Hospital (ethics review board no. 4980) and the ethics committees of the other participating institutions. Owing to the retrospective nature of the study, written informed consent from patients was waived; however, the study protocol was posted on the hospital website, and opt-out consent, obtained.

The diagnostic criteria for CMV-AU were established on the basis of the typical clinical findings described in the literature and PCR results of AH [10, 27]. Therefore, patients diagnosed with CMV-AU presented with most (but not necessarily all) of the following clinical symptoms: small to medium white keratic precipitates (KPs), (mainly categorized in coin-shaped, mutton-fat, stellate, and dendritic patterns), corneal edema, Descemet membrane folds (DFs), anterior chamber (AC) cells, iris atrophy, elevated IOP, and positive PCR results for CMV-DNA. In this study, patients with unilateral anterior segment inflammation showing the above ocular findings and recurrent episodes of elevated IOP were included even if the PCR assays of AH were negative for CMV-DNA. The exclusion criteria were positive syphilis serology, signs of sarcoidosis, and positive PCR results for herpes simplex virus (HSV) or varicella-zoster virus (VZV) in AH. Patients with CMV retinitis were also excluded.

Two percent GCV eye drops were prepared by dissolving GCV intravenous infusion 500 mg in physiologic saline to a 2.0% concentration and filtering the solution through a 0.22-μm membrane filter. Regarding the use and adverse event survey of 2% GCV eye drops as an in-house preparation, approval was obtained from the Unapproved/New Drug Evaluation Committee of the National Defense Medical College Hospital.

Betamethasone sodium phosphate 0.1% ophthalmic solution was used as the topical corticosteroid. Generally, instillation was initiated at a frequency of 4 times daily and subsequently adjusted as needed according to the clinical signs and symptoms. Recurrence was defined as the presence of inflammatory cells in the AC or elevation of IOP, or both. If relapse occurred after tapering the 2% GCV eye-drop treatment and could not be resolved by increasing the ongoing therapy, systemic GCV was administered, followed by a switch back to topical administration after the inflammation subsided. Automated static perimetry was performed in cases with persistent elevated IOP during follow-up at our institution. Glaucoma was defined as the presence of visual field defects consistent with changes in the optic nerve head findings.

Statistical analysis was performed with JMP Pro version 17 (Business Unit of SAS). The Wilcoxon signed rank test for paired nonparametric data and the chi-square test for categorical variables were used. Probability values less than. 05 were considered significant.

## Result

Table 1 shows the background of the patients diagnosed with CMV-AU and treated with 2% GCV eye drops. The mean age was 63.4 ± 14.1 years, the male-to-female ratio was 11:1, and all the patients had unilateral CMV-AU. The mean follow-up period at the referring clinic was 33.2 ± 42.3 months, and the most common subjective symptom was blurred vision (10 of 12 patients, 83.3%), followed by decreased visual acuity (2 patients, 16.7%). The most common referral reason was unidentified uveitis (66.7%), followed by high IOP including glaucoma (50.0%) and PSS (33.3%). Only 2 patients (16.7%) were referred because of herpetic anterior uveitis. All the patients with a history of ocular hypertension were prescribed antiglaucoma eye drops. Additionally, all but 1 patient (91.7%) received corticosteroid eye drops.

**Table 1:** Background of patients with cytomegalovirus-associated anterior uveitis

	Patients with CMV-AU (N = 12)
Age, y	63.4 ± 14.1* (68.5, 38–81)^†^
Male/Female	11/1
Affected eye, n (%)	
Unilateral	9 (100)
Bilateral	0 (0)
Subjective symptoms, n (%)	
Blurred vision	10 (83.3)
Decreased visual acuity	2 (16.7)
Referral reasons^§^, n (%)	
Unidentified uveitis	8 (66.7)
Glaucoma including high IOP	6 (50.0)
Posner-Schlossman syndrome (PSS)	4 (33.3)
Herpetic anterior uveitis	2 (16.7)
History of ocular hypertension or glaucoma	10 (83.3)
Treatment at referral clinic, n (%)	
Corticosteroid eye drops	11 (91.7)
Antiglaucoma eye drops	11 (91.7)

*CMV* cytomegalovirus, *AU* anterior uveitis, *logMAR VA* logarithm of the minimum angle of resolution visual acuity, *IOP* intraocular pressure

^*^Mean ± SD

^†^Median, range

^§^Including overlaps

Table 2 shows the ocular findings of individual patients with CMV-AU at the initial presentation and the aqueous humor PCR results. BCVA worse than 1.0 was observed in 4 eyes (33.3%), and of 0.1 or worse, in 2 eyes (16.7%). Elevated IOP of 21 mm Hg or higher was observed in 6 eyes (50.0%). KPs were present in 7 eyes (58.3%) including 2 eyes (16.7%) with coin-shaped KPs, and DFs were not observed in any of the eyes. Corneal edema, anterior chamber (AC) cells, and iris atrophy were observed in 4 eyes each (33.3%). CMV DNA was detected in the initial AH PCR in 8 patients (66.7%), and ultimately, repeated AH PCR conducted at the time of recurrence yielded positive results in 10 patients (83.3%). All the patients were treated with at least 2 different antiglaucoma medications. None had a history of glaucoma surgery. Regarding case 2, the patient had a history of rheumatoid arthritis and was receiving immunosuppressive medication. The other patients had no underlying conditions associated with immunodeficiency.

**Table 2:** Ocular findings at the first visit and aqueous humor PCR results in individual patients with cytomegalovirus-associated anterior uveitis

Case	Age,y	Sex	BCVA	IOP, mm Hg	KPs	DFs	Coin lesion	AC cells	Iris atrophy	Pseudophakia	1st PCR result	Final PCR result	Copy number of CMV DNA
1	60	M	1.5	27.3	+	−	−	−	+	−	−	+	1.9×10^5
2	78	F	1	27	−	−	−	−	−	−	+	+	7.0×10^5
3	68	M	0.6	16	+	−	−	−	−	−	+	+	5.3×10^4
4	49	M	1	13.5	+	−	−	−	−	−	+	+	1.1×10^5
5	81	M	HM	35.5	−	−	−	−	−	+	+	+	5.5×10^6
6	71	M	1	20.3	+	−	−	+	−	+	+	+	2.2×10^6
7	71	M	0.1	16	−	−	−	+	−	−	−	+	8.2×10^4
8	47	M	1.5	28	−	−	−	+	+	−	+	+	7.7×10^6
9	78	M	0.7	22	−	−	−	−	+	+	+	+	4.1×10^5
10	51	M	1.5	17	+	−	−	+	−	−	−	−	−
11	69	M	1.2	12.3	+	−	+	−	+	−	+	+	2.2×10^5
12	38	M	1.5	40	+	−	+	−	−	−	−	−	−

*BCVA* best-corrected visual acuity, *IOP* intraocular pressure, *KPs* keratic precipitates, *DFs* Descemet membrane folds, *AC* anterior chamber, *PCR* polymerase chain reaction, *HM* hand motions

**Fig. 1:**
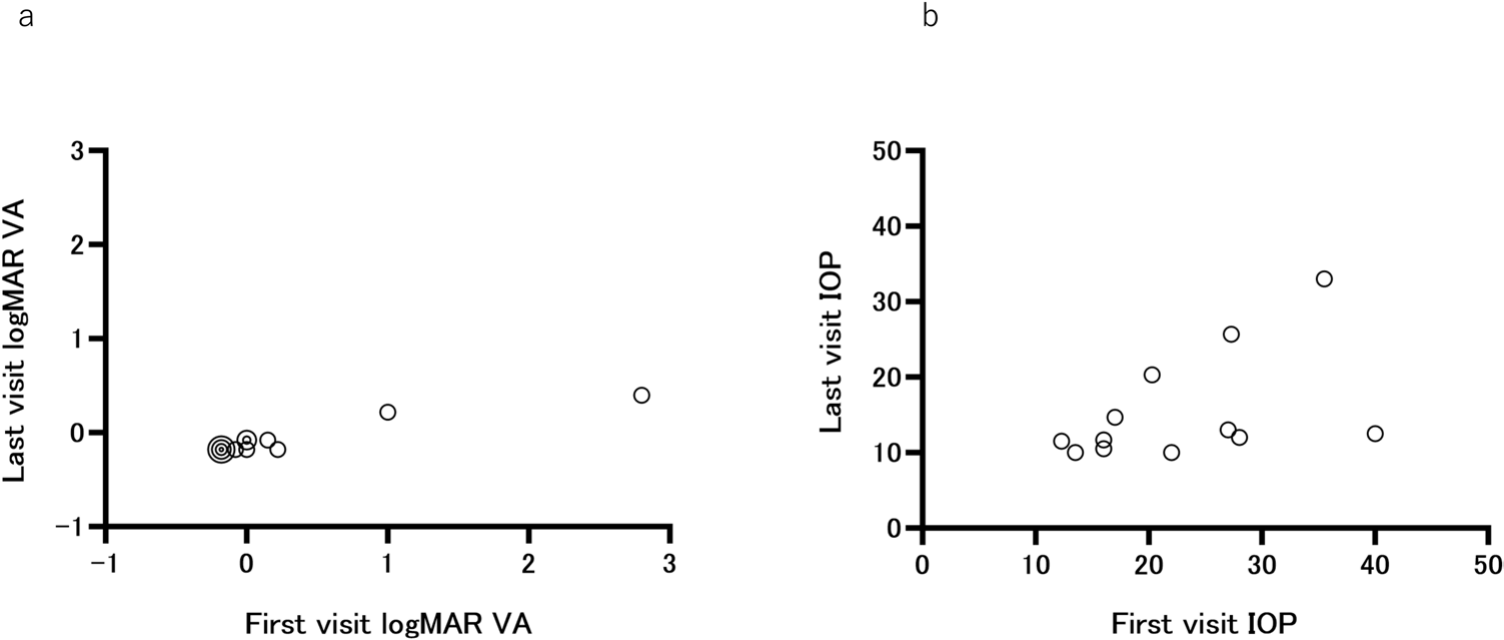
Changes in logMAR VA and IOP between the initial and final visits. Scatterplots of **a** logMAR VA and **b** IOP at the initial and final visits. Cases are represented by circles. In **a**, when case plots completely overlap, circles corresponding to the number of cases are overlaid for clarity. A quadruple circle represents 4 cases, and a double circle represents 2 cases

The clinical course of each CMV-AU patient is shown in Table 3, and the overall analysis results are presented in Table 4. The mean follow-up period was 19.5 ± 12.8 months, and the mean treatment period with 2% GCV eye drops, 11.9 ± 10.0 months. Topical corticosteroids and antiglaucoma eye drops were continued in 11 patients and initiated in the remaining patient (case 12). In 5 of the 12 patients (41.7%), oral GCV was temporarily administered in conjunction with 2% GCV eye drops for recurrent uveitis or elevated IOP, but none of the patients received intravenous GCV. Recurrence of uveitis was observed in 66.7% of the patients, and recurrence of uveitis per year was 1.01. Glaucoma developed in all the patients except the patient of case 12, who had the shortest overall disease duration. Trabeculectomy was performed in case 7. Table 5 shows the ocular findings of individual patients with CMV-AU at the last presentation. BCVA higher than 1.0 was observed in 10 patients (83.3%), and no cases had BCVA of 0.1 or lower. IOP of 21 mm Hg or higher was present only in 2 eyes (16.7%). As shown in Figure 1, no patients showed worsening of BCVA (Fig. 1a) or IOP (Fig. 1b). KPs, including coin-shaped lesions or DFs, were not observed in any of the eyes. AC cells were present in 3 eyes (25%), and iris atrophy was noted in 4 eyes (33.3%). The improvement in BCVA is likely attributable to the resolution of anterior chamber inflammation with treatment and to the subsequent reduction in IOP leading to the improvement of corneal edema. Corticosteroid eye drops were continued in all the cases except case 8, but antiglaucoma eye drops were discontinued in 6 of the 12 cases.

**Table 3:** Clinical course of individual patients with cytomegalovirus-associated anterior uveitis

Case	Follow-up period at the referral clinic, mo	Follow-up period from first visit, mo	Treatment period of 2% GCV eye drops, mo	Corticosteroid eye drops	Antiglaucoma eye drops	Oral GCV	GCV infusion	Recurrence of uveitis	Glaucoma	TLE
1	3	24	21	+	+	−	−	2	+	−
2	3	29	6	+	+	−	−	0	+	−
3	70	30	13	+	+	−	−	3	+	−
4	140	20	19	+	+	+	−	2	+	−
5	41	18	6	+	+	−	−	0	+	−
6	1	39	37	+	+	+	−	1	+	−
7	28	39	14	+	+	+	−	1	+	+
8	17	3	4	+	+	−	−	0	+	−
9	72	7	6	+	+	+	−	1	+	−
10	2	5	4	+	+	+	−	1	+	−
11	21	11	11	+	+	−	−	3	+	−
12	0	9	2	+	+	−	−	0	−	−

*GCV* ganciclovir, *TLE* trabeculectomy

**Table 4:** Overall analysis of clinical course of patients with cytomegalovirus-associated anterior uveitis

	Patients with CMV-AU (n = 12)
Follow-up period at the referral clinic, mo	33.2 ± 42.3* (19, 0–140)^†^
Follow-up period from first visit, mo	19.5 ± 12.8 (19, 3–39)
Treatment period of 2% GCV eye drops, mo	11.9 ± 10.0 (8.5, 2–37)
Corticosteroid eye drops	12 (100^§^)
Antiglaucoma eye drops	12 (100)
Oral GCV	5 (41.7)
GCV infusion	0 (0)
Recurrence of uveitis	8 (66.7)
Recurrence of uveitis per year	1.01
Glaucoma	11 (91.7)
Trabeculectomy	1 (8.3)

*CMV* cytomegalovirus, *GCV* ganciclovir

^*^Mean ± SD

^†^Median, range

^§^Values are presented as percentages

**Table 5:** Ocular findings in individual patients with cytomegalovirus-associated anterior uveitis at the last visit

Case	BCVA	IOP, mm Hg	KPs	Coin-shaped KPs	DFs	AC cells	Iris atrophy	Corticosteroid eye drops	Antiglaucoma eye drops
1	1.5	25.7	−	−	−	−	+	+	+
2	1.2	13	−	−	−	−	−	+	+
3	1.5	10.5	−	−	−	−	−	+	−
4	1.2	10	−	−	−	−	−	+	+
5	0.4	33	−	−	−	−	−	+	+
6	1.5	20.3	−	−	−	−	−	+	+
7	0.6	11.7	−	−	−	+	−	+	−
8	1.5	12	−	−	−	−	+	−	+
9	1.2	10	−	−	−	−	+	+	+
10	1.5	14.7	−	−	−	+	−	+	−
11	1.5	11.5	−	−	−	+	+	+	+
12	1.5	12.5	−	−	−	−	−	+	−

*BCVA* best-corrected visual acuity, *IOP* intraocular pressure, *KPs* keratic precipitates, *DFs* Descemet membrane folds, *AC* anterior chamber

Table 6 shows the comparison of logMAR VA, IOP, and corneal endothelial cell counts at the initial and final visits of the CMV-AU patients who received 2% GCV eye-drop treatment. The mean logMAR VA was 0.27 ± 0.83 at the initial visit and -0.07 ± 0.19 at the final visit, showing a slight improvement, but the difference was not significant. However, IOP significantly decreased from the mean of 22.9 ± 8.8 mm Hg at the initial visit to 15.4 ± 7.2 mm Hg at the final visit. The mean corneal endothelial cell count was 2243 ± 692 at the initial visit and 2079 ± 879 at the final visit, showing minimal decrease without statistical significance.

**Table 6:** Comparison of logMAR VA, IOP, and corneal endothelial cell count at the initial and final presentations

	First visit	Final visit	*P* value^§^
LogMAR VA	0.27 ± 0.83* (0, − 0.18–2.70)^†^	− 0.07 ± 0.19 (− 0.18, − 0.18–0.40)	0.1277
IOP, mm Hg	22.9 ± 8.8 (21.2, 12.3–40)	15.4 ± 7.2 (12.3, 10–33)	0.0086
Corneal endothelial cell count, cells/mm^2^	2243 ± 692 (2291, 1368–3021)	2079 ± 879 (2318.5, 822–2857)	0.7728

*LogMAR VA* logarithm of the minimum angle of resolution visual acuity, *IOP* intraocular pressure, *KPs* keratic precipitates, *DFs* Descemet membrane folds, *AC* anterior chamber

^*^Mean ± SD

^†^Median, range

^§^Wilcoxon signed rank test

## Discussion

In line with previous studies [5, 28, 29], our CMV-AU cohort exhibited a significant male predominance and unilateral disease presentation. After initiation of 2% GCV eye drops, all the patients demonstrated positive therapeutic responses, including improved BCVA, decreased IOP, and resolution of KPs, including coin-shaped lesions. Furthermore, the treatment led to a reduction in the need for antiglaucoma medications and to maintenance of corneal endothelial cell density, with no patients developing bullous keratopathy. No serious adverse events were observed, and all the patients continued the treatment, suggesting a high safety profile for 2% GCV eye drops.

The patients had undergone a prolonged mean follow-up period of 33.2 ± 42.3 months at referral clinics. Except for the patient of case 12, who had a short duration of CMV-AU, all the patients were treated with topical corticosteroids and antiglaucoma eye drops for presumed idiopathic uveitis and secondary glaucoma including high IOP. This is because the ocular inflammation in CMV-AU is mild and the peak of IOP can be lowered with medications. Also, the ocular findings are similar to PSS and Fuchs uveitis syndrome but lack characteristic findings, which is why the referring diagnosis of herpetic iridocyclitis was low, at 16.7%.

AH PCR demonstrated final CMV-DNA positivity in 83.3% (10/12) of the cases, highlighting its value in the early diagnosis and management of CMV-AU. However, the positive rate of CMV-DNA in AH PCR at the initial visit was 66.7% (8/12). Even in the 4 patients with negative initial AH PCR results, KPs or AC cells were observed at the initial visit. Two of these patients experienced recurrence after the initiation of 2% GCV eye drops, and CMV-DNA became positive in AH PCR for CMV-DNA at the time of recurrence. These findings suggest that when CMV-AU is suspected on the basis of the clinical course, it is important not to make a diagnosis based on the result of a single AH PCR test, but to repeat the test at the time of recurrence.

Among the 2 patients (cases 10 and 12) with negative AH PCR results, both initially presented with granulomatous uveitis and were started on topical corticosteroids; however, the inflammation did not improve. Although the AH PCR was negative, a coin lesion was observed at the follow-up visit, leading to the suspicion of CMV-AU. Oral ganciclovir was initiated for diagnostic and therapeutic purposes, which resulted in resolution of the inflammation. After 1 week of oral treatment, the medication was switched to 2% GCV eye drops, and the inflammation remained quiescent. No recurrence of inflammation has been observed thereafter in case 12. However, recurrence was observed in case 10 owing to poor adherence and self-reduced frequency of eye-drop administration.

Additionally, the presence of "coin-shaped KPs," characterized by annular, dust-like KPs, is a hallmark pathologic feature of CMV endotheliitis, which demonstrated a high positivity rate of 60% to 70% [30, 31]. However, only 2 patients (16.7%) exhibited a coin lesion in this study. Coin-shaped KPs are an uncommon finding in CMV-AU, with a reported prevalence of less than 50% in other studies [32]. In addition, although the presence of coin-shaped KPs was correlated with high copy numbers (10^5^–10^3^ copies/mL) of the CMV DNA [33], a high copy number of CMV DNA (2.2 x 10^5^ copies) was detected by AU PCR in case 11, whereas CMV DNA was not detected in case 12.

Recurrence of CMV-AU under treatment with 2% GCV has been reported in several studies. Chen and colleagues reported a recurrence rate of 44% (17 of 38 eyes) over long-term follow-up periods averaging 45.6 months, with a recurrence rate of 0.13 relapses per year [34]. Keorochara and colleagues observed recurrence in 6 of 11 cases (54.5%) within a 1-year follow-up period. Notably, in 3 of those cases, 2% GCV eye drops had been discontinued, indicating that discontinuation or cessation of 2% GCV eye drops is a risk factor for recurrence of uveitis [35]. In this study, recurrence was observed in 8 of 12 cases (66.7%), with an average of 1.17 ± 1.11 relapses per patient and an average of 0.62 relapses per year. This recurrence rate was marginally higher than those in previous reports. Although all the CMV-AU patients were under treatment with 2% GCV eye drops, the possibility that poor adherence to eye-drop administration contributed to the recurrence cannot be ruled out. Since GCV is a viral replication inhibitor and does not eradicate latent virus, maintenance therapy may be required to prevent recurrence. On the other hand, recurrences were controlled with an increase in topical corticosteroids or antiglaucoma eye drops, or both, or with short-term oral GCV, with no recurrences observed in the remaining 33.3% of cases, indicating efficacy of topical 2% GCV in both preventing recurrences and minimizing the need for systemic therapy. This finding was consistent with those of previous reports [32, 35–37].

A marked decrease in corneal endothelial cell density can be a significant indicator of corneal endotheliitis associated with CMV [38]. The corneal endothelial cell count decreased slightly, from 2243 ± 692 cells/mm^2^ to 2079 ± 879 cells/mm^2^, after the initiation of 2% GCV eye drops. However, this difference was not significant, which is consistent with the findings of previous reports using 2% GCV [30, 36]. This result could be attributable to the effect of 2% GCV eye drops. However, further studies with a larger number of cases and longer follow-up periods are needed, given the small sample size and the short mean follow-up period of 19.5 ± 12.8 months in this study.

Mori and colleagues reported that 47.1% of patients who received antiviral therapy, including 0.5% GCV eye drops, underwent glaucoma surgery [39]. In the current study, only 1 patient (case 7) received concomitant oral GCV and subsequently required further surgical intervention with trabeculectomy. However, this patient showed no episodes of IOP spikes after trabeculectomy, allowing for discontinuation of antiglaucoma eye drops, which suggests that increasing the concentration of GCV eye drops to 2% may reduce the need for glaucoma surgery. Touhami and colleagues suggested that earlier initiation of antiviral therapy (within or before 700 days) may decrease the need for glaucoma surgery [27]. In the case that required trabeculectomy in this study, the follow-up period at the referral clinic and that from the first visit without 2% GCV eye drops was 53 months, suggesting that prolonged follow-up without antiviral therapy may increase the risk of glaucoma surgery.

GCV gel reportedly had moderate response rates for CMV-AU, but its recurrence rates were lower than those of systemic and intravitreal GCV [40]. However, in the study by Boonhaijaroen and colleagues, systemic GCV demonstrated a significant advantage over topical GCV in terms of achieving a more rapid therapeutic response, with improvements observed within approximately 1 month and 2 months, respectively [41]. However, most patients in the topical group continued medication with few attempts to discontinue it, whereas patients in the systemic group requested discontinuation. In the current study, all the patients continued to use 2% GCV eye drops without any reported adverse effects, which suggests a high safety profile.

Epidemiologic studies on the causes of uveitis in Japan were conducted in 2002, 2009, and 2016, led by the Japanese Ocular Inflammation Society [42–44]. Because CMV-AU in immunocompetent individuals was recognized after 2005 owing to the widespread use of AH PCR [3, 13], the 2002 statistics do not include CMV-AU. In herpetic AU, the rate of AH PCR testing and the detection rate of CMV-DNA in PCR-tested cases were 46.5% and 27.0% in 2009 and 55.6% and 48% in 2016, respectively. Whilst CMV was the third most common cause of herpetic AU after herpes simplex virus (HSV) and varicella zoster virus (VZV) in 2009, it became the most common cause, followed by VZV and HSV, in 2016. With the further popularization of AH PCR and the progression of the aging society with a declining birthrate, the number of CMV-AU cases is expected to increase further.

This study has several limitations. Its retrospective design introduced potential bias related to the 2% GCV eye-drop protocol. The criteria for evaluating lesion remission and recurrence efficacy were not clearly defined. In cases with CMV detected in the AH PCR, whether 2% GCV eye-drop treatment resulted in a negative CMV PCR in the AH remained unverified. The inhibitory effect of the eye drops on corneal endothelial cell density decrease could not be assessed in all the patients. As a single-center study, controlling for potential confounding variables was challenging, potentially biasing data collection. The small sample size (N = 12) limits the generalizability of the findings to the broader CMV-AU population. The absence of a control group makes it difficult to definitively attribute observed improvements solely to the 2% GCV eye drops. The heterogeneity of adjunctive therapies (corticosteroids, antiglaucoma medications, oral GCV) may have confounded the evaluation of the 2% GCV ophthalmic solution's efficacy.

In conclusion, topical 2% GCV eye drops effectively suppressed CMV-AU recurrence, reducing the need for systemic GCV. Furthermore, by controlling IOP, it contributed to the management of secondary glaucoma. No adverse events related to 2% GCV eye drops were observed during the follow-up period, suggesting a favorable safety profile. Long-term topical administration of 2% GCV is recommended for the prevention of recurrence.
